# Appointment of Dr. E. N. Horsford

**Published:** 1850-11

**Authors:** 


DR. E. N. HORSFORD has been appointed Professor of Chemistry in Massachusetts Medical College, in place of Dr. Webster.

